# Supplementary material for: Structural characterisation of the fungal Pmt4 homodimer
Source: Nat Commun. 2025 Dec 14;16:11134. doi: 10.1038/s41467-025-67412-1 (PMC12705809; doi:10.1038/s41467-025-67412-1)
Supplement: Supplementary file 1 — Supplementary Information [file 41467_2025_67412_MOESM1_ESM.pdf]

Supplementary Figures

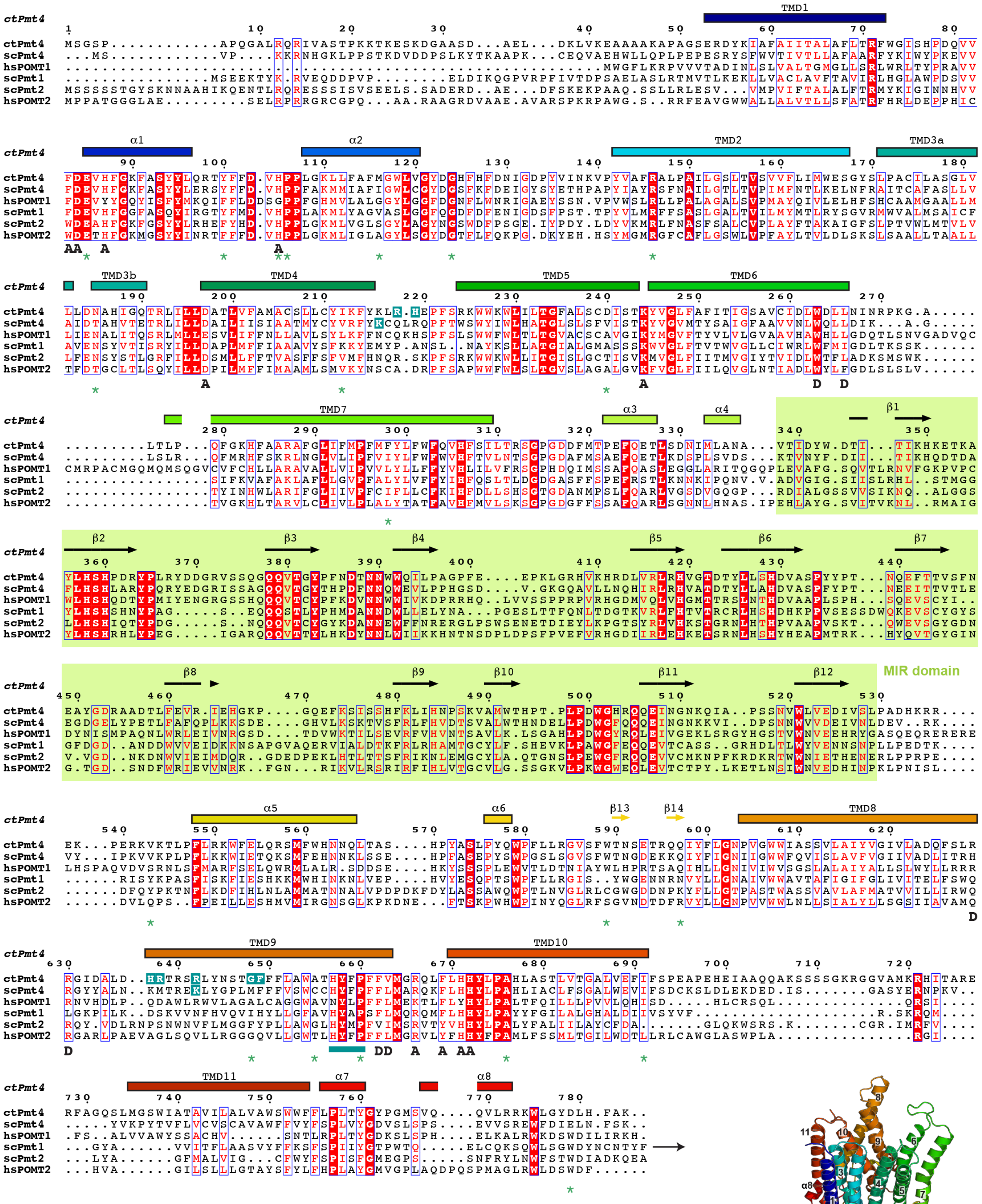

Percent identity matrix

	ctPmt4	scPmt4	hsPOMT1	scPmt1	scPmt5	scPmt2	scPmt3	scPmt6	hsPOMT2
ctPmt4	100	48	33	30	27	29	30	29	32
scPmt4	48	100	31	31	26	28	27	28	29
hsPOMT1	33	31	100	29	28	30	31	29	33
scPmt1	30	31	29	100	34	34	32	32	33
scPmt5	27	26	28	34	100	30	28	27	28
scPmt2	29	28	30	34	30	100	66	45	35
scPmt3	30	27	31	32	28	66	100	45	34
scPmt6	29	28	29	32	27	45	45	100	33
hsPOMT2	32	29	33	33	28	35	34	33	100

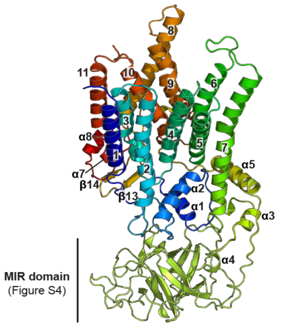

Supplementary Fig. 1. Sequence alignment of PMT homologues

Sequence alignment for PMT homologues generated using Clustal Omega[58] and visualised using ESPript 3.0[59]. Sequences are displayed from *Chaetomium thermophilum* (ct), *Saccharomyces cerevisiae* (sc) and *Homo sapiens* (hs). Conserved residues involved in interactions at the *ct*Pmt4 homodimer interface (D) or with substrate/product in the active site (A) are indicated. Dol-P-Man binding residues in *ct*Pmt4 and functionally important residues in the cytosolic pocket of *sc*Pmt4 are highlighted in teal, whilst the conserved HYΦP motif within the putative flippase channel is shown by a teal bar below the alignment. Secondary structure features present within our model for *ct*Pmt4 are coloured from the N- (blue) to the C- (red) terminus according to the cartoon representation (bottom right). POMT1 residues within the TMDs causing human pathologies are shown by green asterisks below the alignment. The sequence of the MIR domain is highlighted in green and annotated in more detail in Supplementary Fig. 5. The percent identity matrix (bottom left) includes all *S. cerevisiae* homologues, and PMT4 (orange), PMT1 (green) and PMT2 (blue) members are coloured accordingly.

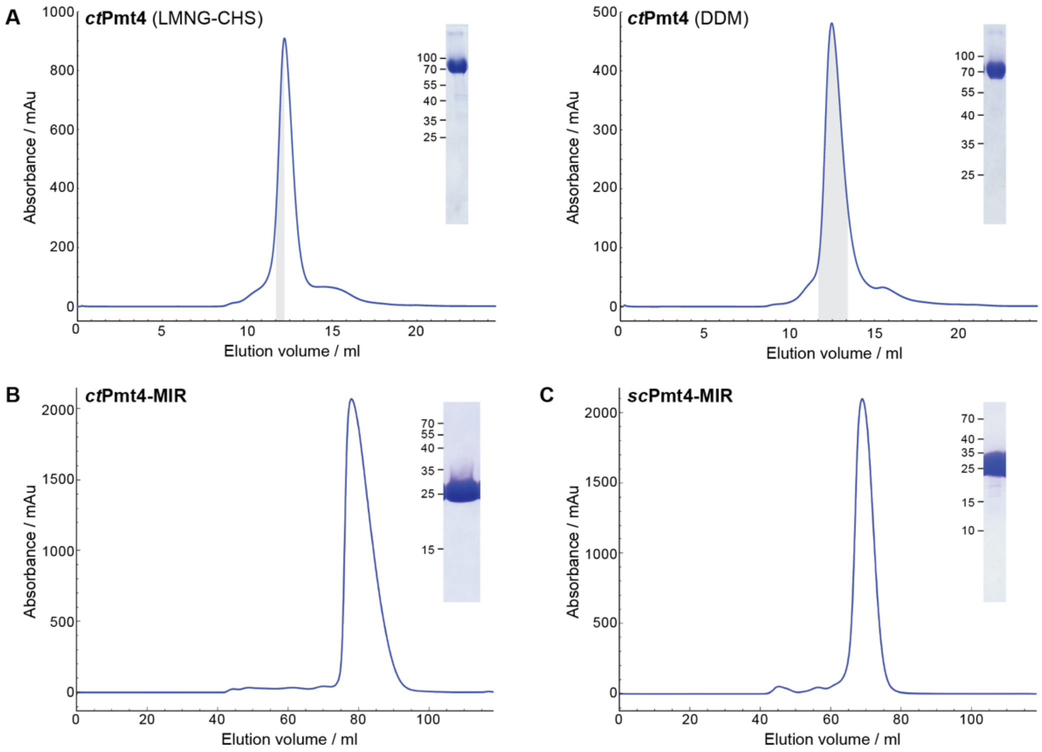

Supplementary Fig. 2. Purification of Pmt4 full-length and MIR domain constructs

Size exclusion chromatography profiles for the indicated constructs. The insets show Coomassie-stained SDS-PA gels for the final samples. **A)** Full-length *ct*Pmt4 was purified in LMNG-CHS for cryo-EM (left) and in DDM for native MS (right), with the fraction(s) taken for each experiment highlighted in grey. The **B)** *ct*Pmt4- and **C)** *sc*Pmt4-MIR domains were purified for X-ray crystallography. These data are representative of at least three independent purifications. Source data are provided in the Source Data file.

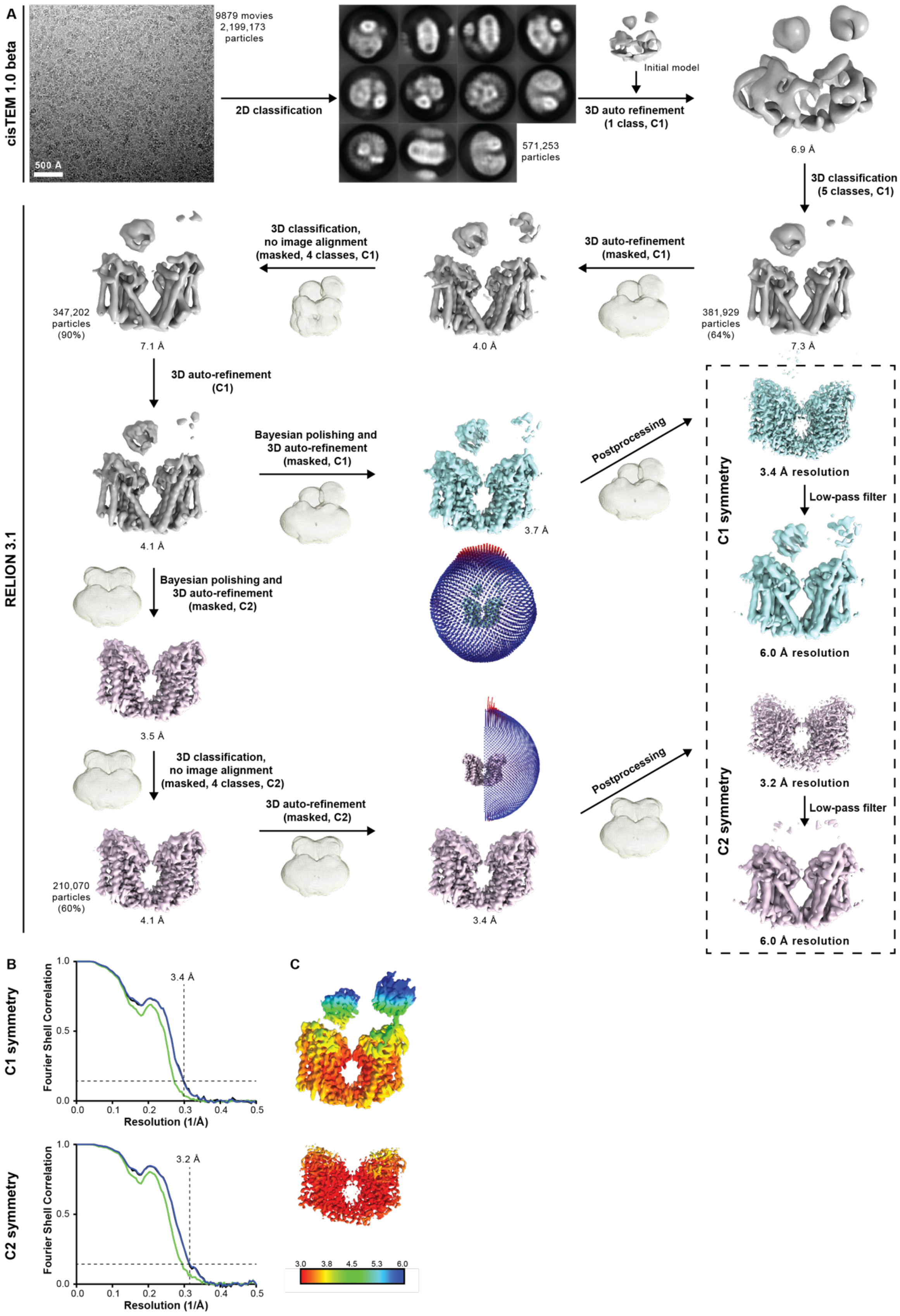
A
cisTEM 1.0 beta
9879 movies
2,199,173
particles
500 Å
2D classification
571,253
particles
Initial model
3D auto refinement
(1 class, C1)
6.9 Å
3D classification
(5 classes, C1)
RELION 3.1
3D classification,
no image alignment
(masked, 4 classes, C1)
3D auto-refinement
(masked, C1)
347,202
particles
(90%)
7.1 Å
4.0 Å
381,929
particles
(64%)
7.3 Å
3D auto-refinement
(C1)
Bayesian polishing and
3D auto-refinement
(masked, C1)
Postprocessing
3.4 Å resolution
Low-pass filter
C1 symmetry
4.1 Å
3.7 Å
6.0 Å resolution
Bayesian polishing and
3D auto-refinement
(masked, C2)
3.5 Å
3D classification,
no image alignment
(masked, 4 classes, C2)
3D auto-refinement
(masked, C2)
Postprocessing
3.2 Å resolution
Low-pass filter
C2 symmetry
210,070
particles
(60%)
4.1 Å
3.4 Å
6.0 Å resolution
B
C1 symmetry
Fourier Shell Correlation
Resolution (1/Å)
3.4 Å
C2 symmetry
Fourier Shell Correlation
Resolution (1/Å)
3.2 Å
C
3.0
3.8
4.5
5.3
6.0

Supplementary Fig. 3. Workflow for cryo-EM single particle data processing of *ct*Pmt4

A) Particles were autopicked from 9879 micrographs like that shown using WARP[46], then subjected to 2D classification and an initial round of 3D auto-refinement in cisTEM 1.0 beta[47]. Particle poses were subsequently transferred to Relion 3.1[43] for several rounds of 3D classification and auto-refinement. Bayesian particle polishing was performed, followed by auto-refinement with either C1 (turquoise) or C2 (pink) rotational symmetry applied. 3D classification with no image alignment further improved the C2 reconstruction, resulting in final C1 and C2 reconstructions with an average resolution of 3.4 Å and 3.2 Å respectively (dashed box). More complete density for low resolution regions were present in reconstructions low-pass filtered to 6.0 Å. Angular distribution plots are shown for each reconstruction following the final refinement. **B)** Gold-standard FSC curves for the final reconstructions, with no mask (green), tight mask (blue) and corrected (black) curves shown. **C)** Local resolution estimates of the final reconstructions for each complex. The C1 reconstruction has been locally sharpened using LocScale[48].

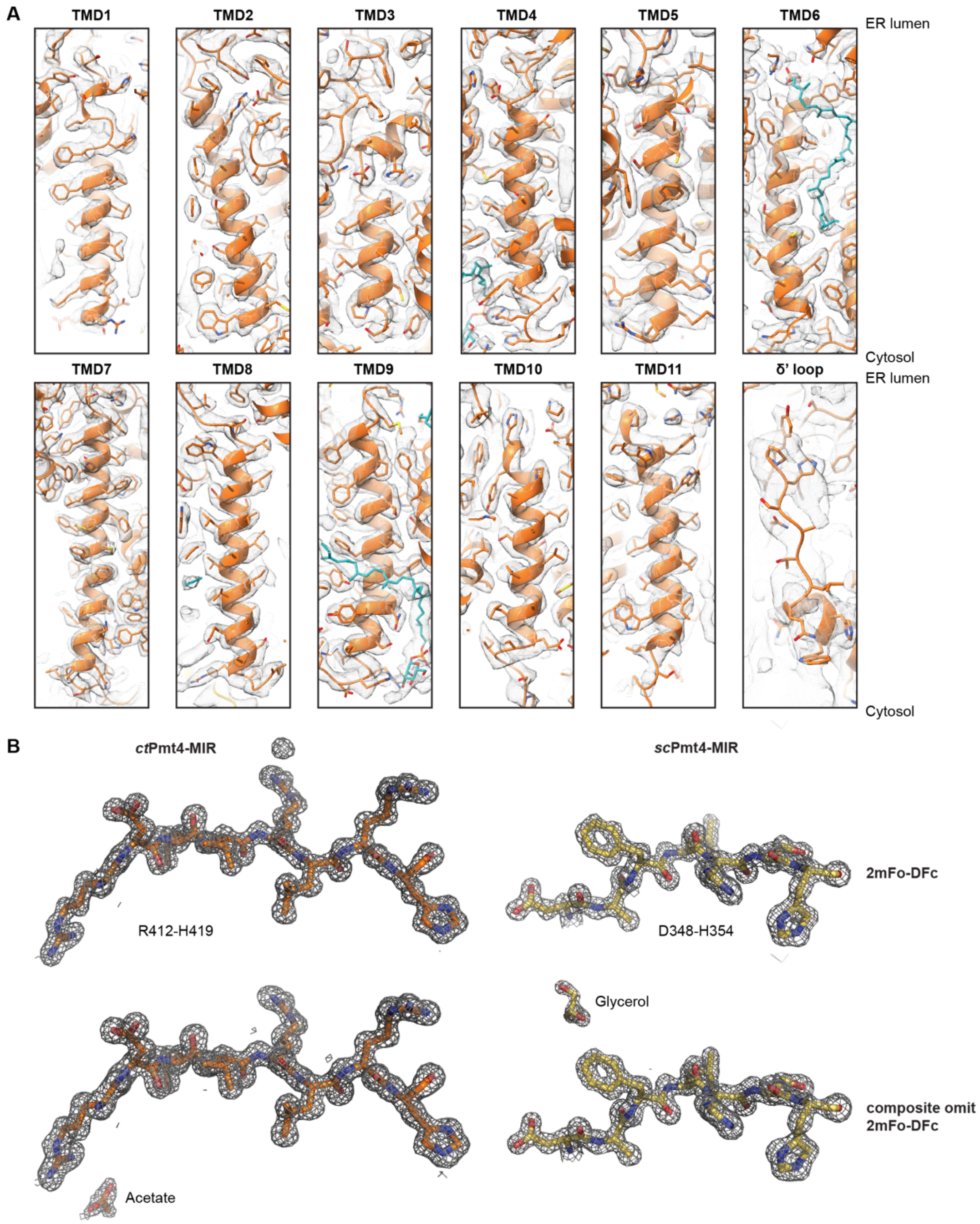

Supplementary Fig. 4. Model to map fits for the Pmt4 structures

A) Density corresponding to *ct*Pmt4 TMDs in the C2 reconstruction. Protein models are shown in orange and lipids in cyan. The map is contoured to 0.016 in all panels, except in the representation of TMD6 where it is contoured to 0.012. The density corresponding to the δ' loop is overlaid with the C2 map low-pass filtered to 6.0 Å and contoured to 0.0055 that was used to assist model building. **B)** Representative electron density from the crystal structures of the Pmt4-MIR domains. All maps are contoured to 1.5σ.

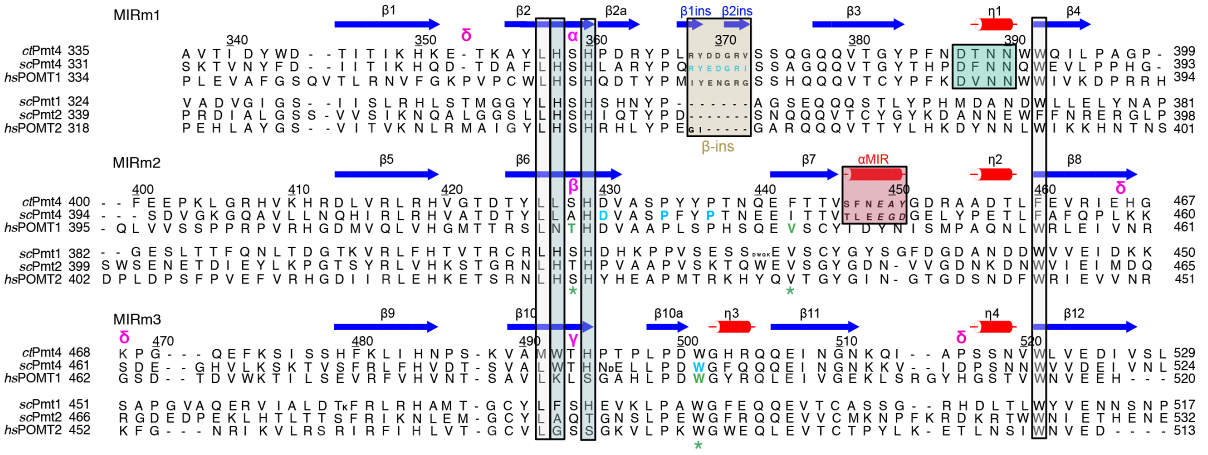

Supplementary Fig. 5. Sequence motifs within PMT-MIR domains

Structure-based alignment of the MIR domains from the PMT homologues in Supplementary Fig. 1. Each line of the alignment corresponds to a MIR-motif and motifs are themselves aligned in order to show sequence conservations, with the hallmark functional histidine and scaffolding leucine/tryptophan residues being highlighted by contiguous blue and grey bars respectively. The DxNN motif is boxed in green, whilst the unique αMIR and β-ins features of PMT4-MIR domains are boxed in red and beige respectively. α to δ sugar binding sites are marked in magenta, residues mutated in this study for in vivo functional assays are shown in blue and POMT1 residues causing human pathologies are shown in green with additional asterisks below the alignment.

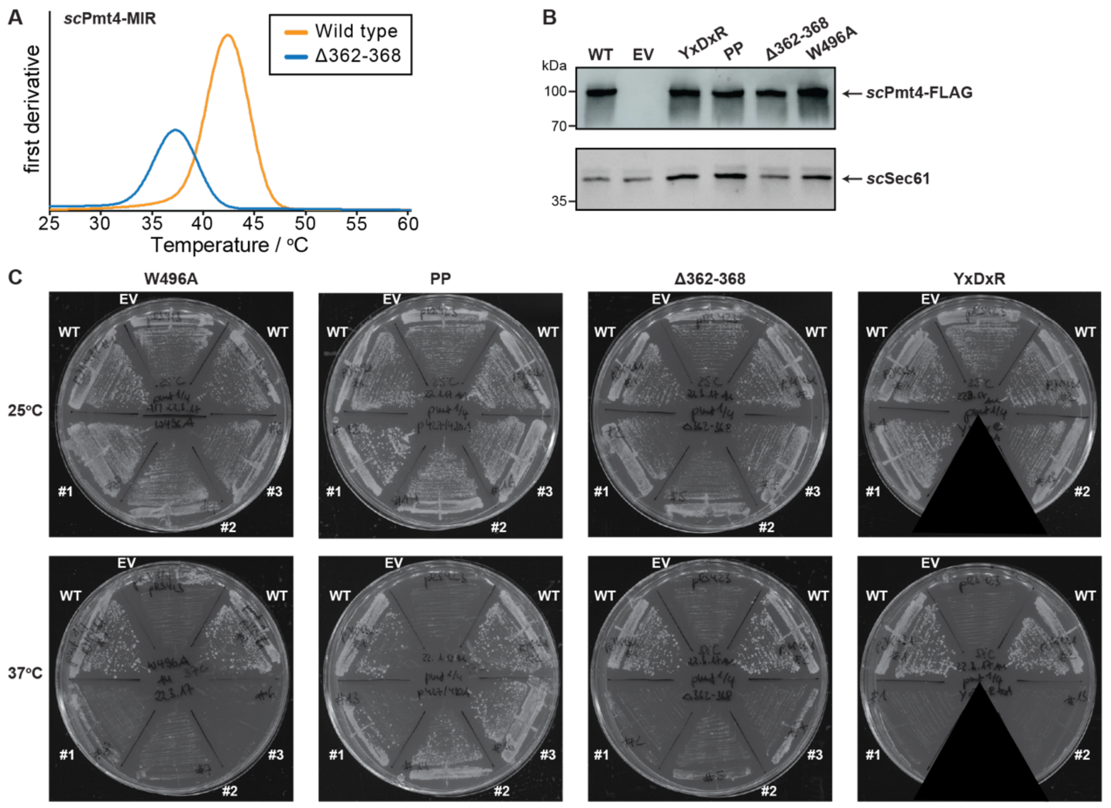

Supplementary Fig. 6. In vivo characterisation of *sc*Pmt4 β-ins mutations

A) nanoDSF measurements of wild-type and Δ362-368 variants of the *sc*Pmt4-MIR domain at 1 mg/ml. Thermogram displays the first derivative of the fluorescence intensity ratio at 350 nm and 330 nm as a function of temperature and is the average of three replicates. The stability as assessed by the melting temperature (T_m) is 42 °C for the wild-type and 37 °C for the Δ362-368 variant. **B)** Western blot of *sc*Pmt4-FLAG isolated from *Δpmt4-CCW5* expressing wild-type *sc*Pmt4 (pJK4-B1; WT), empty pRS423 vector (EV) and β-ins mutants of pJK4-B1 as indicated. 8 µg total membrane protein from each strain was resolved on a 10% SDS-PA gel and probed with anti-FLAG antibody. Sec61 was analysed as a loading control. A representative blot is shown from three independent experiments. Source data are provided in the Source Data file. **C)** Complementation of the temperature-sensitive phenotype of *Δpmt1pmt4*. At least two individual transformants of *Δpmt1pmt4* with pJK4-B1 (WT), empty pRS423 vector (EV) or the indicated β-ins mutants of pJK4-B1 were grown on YPAD plates at 25 °C (top) and 37 °C (bottom) for 3 days.

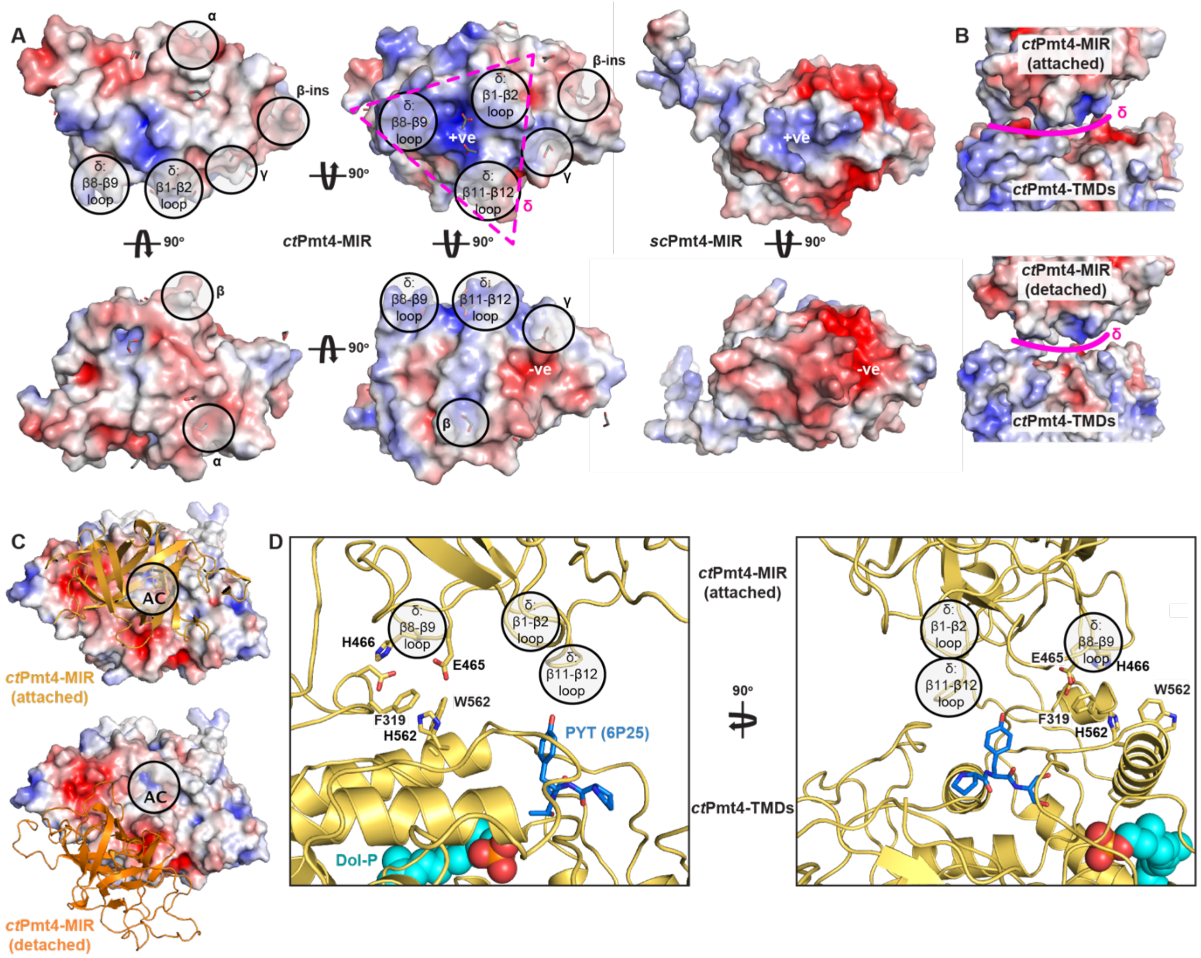

Supplementary Fig. 7. Structural features of Pmt4-MIR domains

A-C) Electrostatic surface potentials (± 5 kT) for different views and interaction surfaces of the Pmt4-MIR domains. **A)** Views of the *ct*Pmt4- and *sc*Pmt4-MIR domains. The upper left *ct*Pmt4 panel corresponds to the view in Fig. 2A (rotated ~30° upwards), whilst the views in the *sc*Pmt4 panels correspond to the right-hand *ct*Pmt4 panels. For *ct*Pmt4, bound acetate and ethylene glycol ligands present in the crystallisation solution are shown as sticks, assigned ligand binding sites (α to δ) are indicated and the positively charged extended δ-site is triangulated in magenta. **B)** The *ct*Pmt4 TMD-MIR interface for the attached and detached MIR domains viewed perpendicular to the membrane plane. **C)** The *ct*Pmt4 TMD-MIR interface for the attached and detached MIR domains viewed from the ER luminal side of the membrane. The MIR domains are shown in cartoon representation and the TMDs in surface representation. The active site (AC) within the TMDs is completely covered by the attached MIR domain. **D)** Detailed views of the TMD-MIR interface for the attached MIR domain. A model substrate (PYT tri-peptide, blue) bound to the *sc*Pmt2[11] (PDB 6P25) is superimposed with the *ct*Pmt4

TMDs as in Fig. 1C to highlight the active site. The loops of the MIR domain δ-site are indicated and residues involved in direct TMD-MIR contacts are labelled.

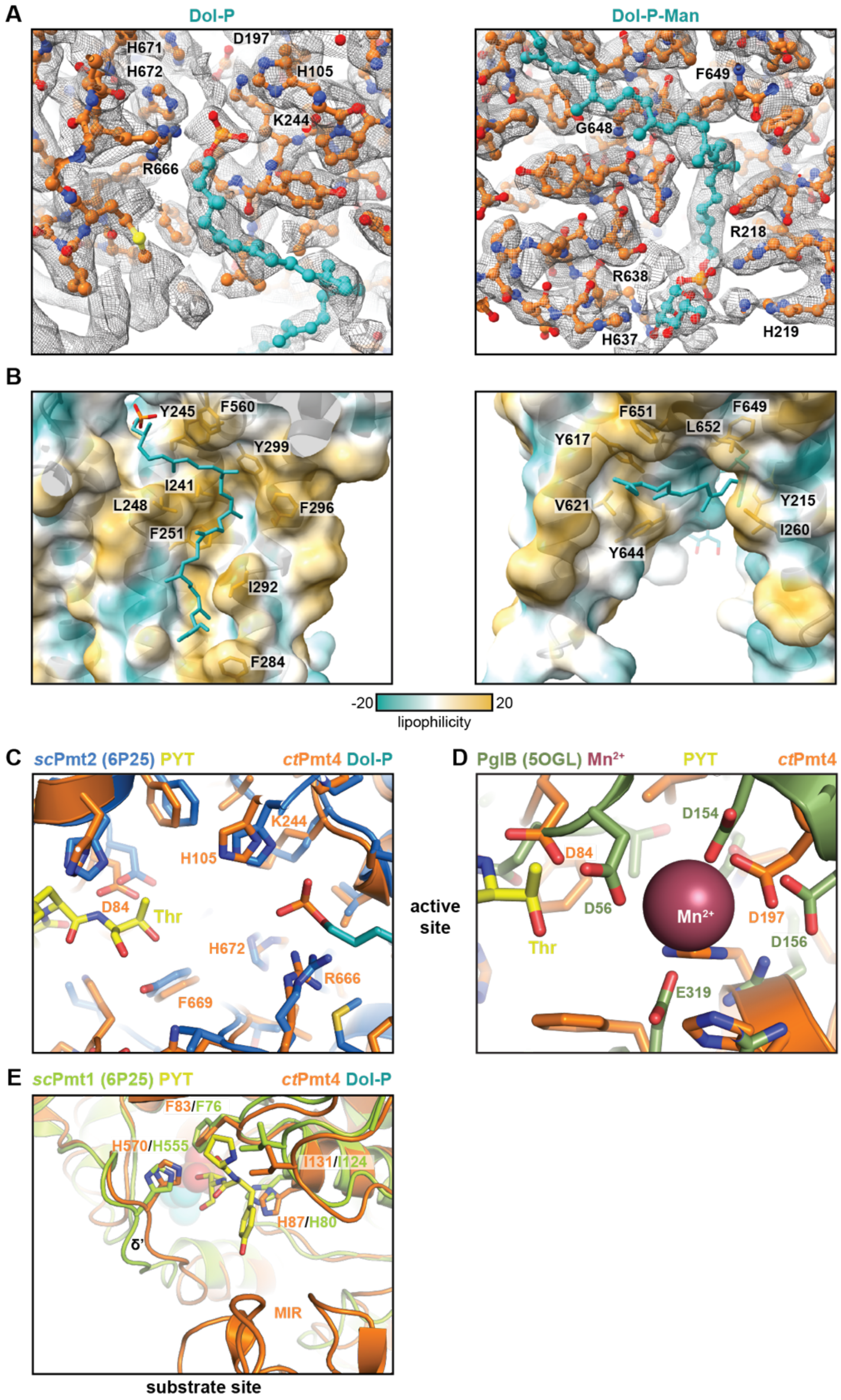

Supplementary Fig. 8. Structural features of dolichol lipid binding sites in *ct*Pmt4

A) Density corresponding to bound dolichol lipids in the C2 *ct*Pmt4 reconstruction. The map is contoured to 0.012 and 0.016 for Dol-P and Dol-P-Man binding sites respectively. Residues involved in binding the lipid headgroups are labelled. The mean correlation coefficient for the ligand fit to map is 0.64. **B)** *ct*Pmt4 TMD binding sites for the dolichol tails of Dol-P and Dol-

P-Man shown in surface representation and coloured according to the molecular lipophilicity potential[60], with hydrophobic interacting residues labelled. **C)** Detailed view of the active sites of *ct*Pmt4 and *sc*Pmt2 shown in the same view as Fig. 3B after superimposition as in Fig. 1C. Conserved residues involved in substrate/product binding are labeled for *ct*Pmt4 and the acceptor threonine of the model substrate bound to *sc*Pmt2 labelled. **D)** Superimposition of the *ct*Pmt4 TMDs with the bacterial oligosaccharyltransferase PglB[21] (PDB 5OGL; RMSD 4.46 Å over 204 Cα-atoms). Mn^{2+} coordinating residues in PglB are labelled, along with putative conserved metal coordinating residues in *ct*Pmt4. The PYT tri-peptide bound to *sc*Pmt2 is also superimposed as in Fig. 1C and the acceptor threonine residue labelled. **E)** Detailed view of the substrate peptide binding sites of *ct*Pmt4 and *sc*Pmt1 shown in the same view as Fig. 3C after superimposition as in Fig. 1C. The PYT tri-peptide bound to *sc*Pmt2 is also superimposed. Corresponding residues proximal to the peptide and the δ' site are labelled. The attached MIR domain of *ct*Pmt4 is shown in cartoon representation.

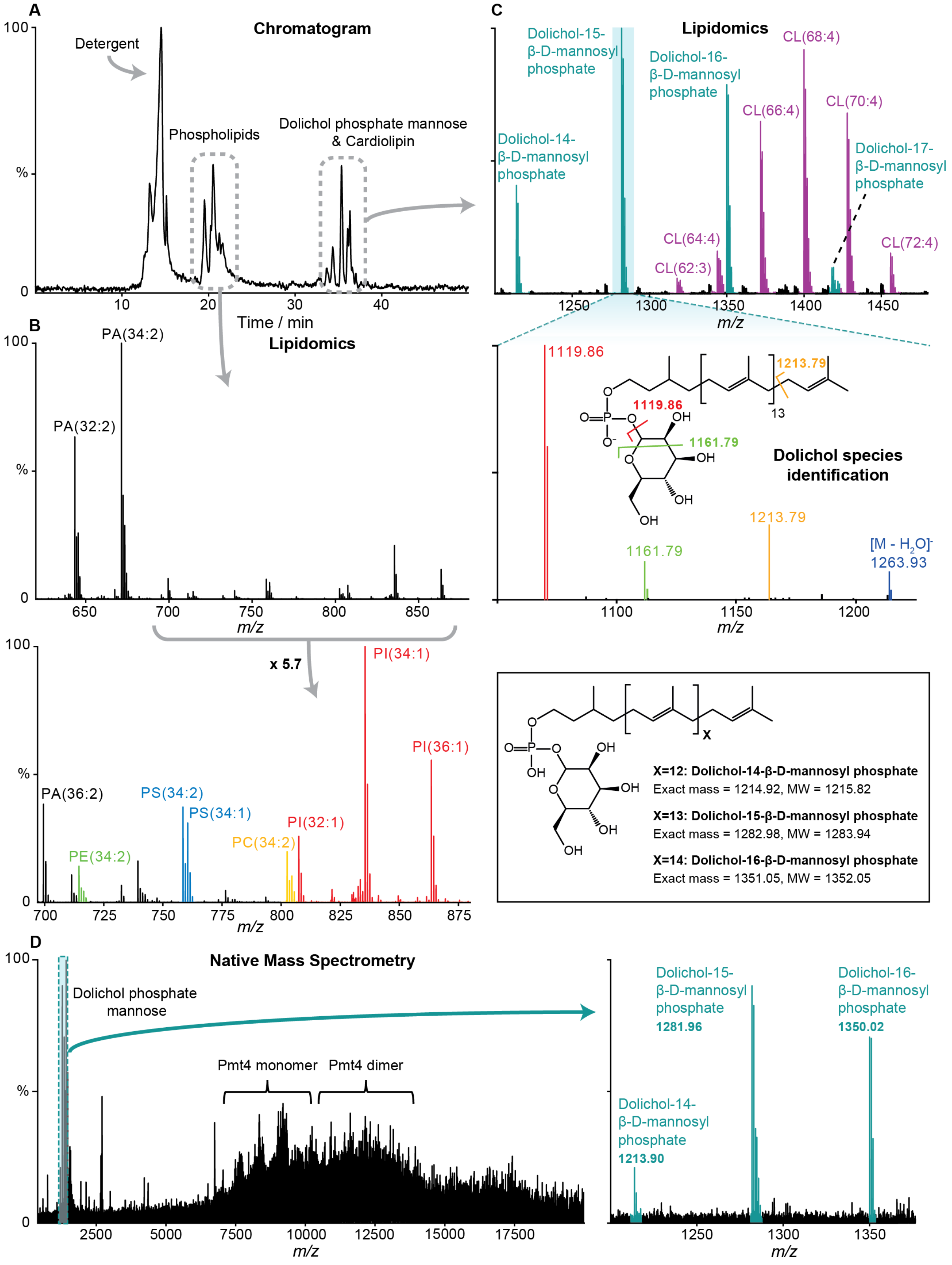

Supplementary Fig. 9. Identification of lipid species co-purified with *ct*Pmt4

A) Total ion chromatogram from lipidomics analysis of full-length *ct*Pmt4 in DDM, obtained after reverse-phased liquid chromatography of trypsin-digested sample. Indicated lipid species

were analysed further by MS. MS profiles of **B)** phospholipid species and **C)** dolichol and cardiolipin species identified through in-solution lipidomics. MS/MS of the highlighted dolichol ion identified the major species associated with the protein as Dol-P-Man. **D)** Mass spectrum of *ct*Pmt4 released from DDM micelles under non-denaturing conditions, acquired using high collision voltage, high resolution (140,000 at *m/z* = 200) and optimised low-mass ion transmission. These conditions enabled detection of dissociated Dol-P-Man, whose molecular weight matched the lipidomics data shown in **C**.

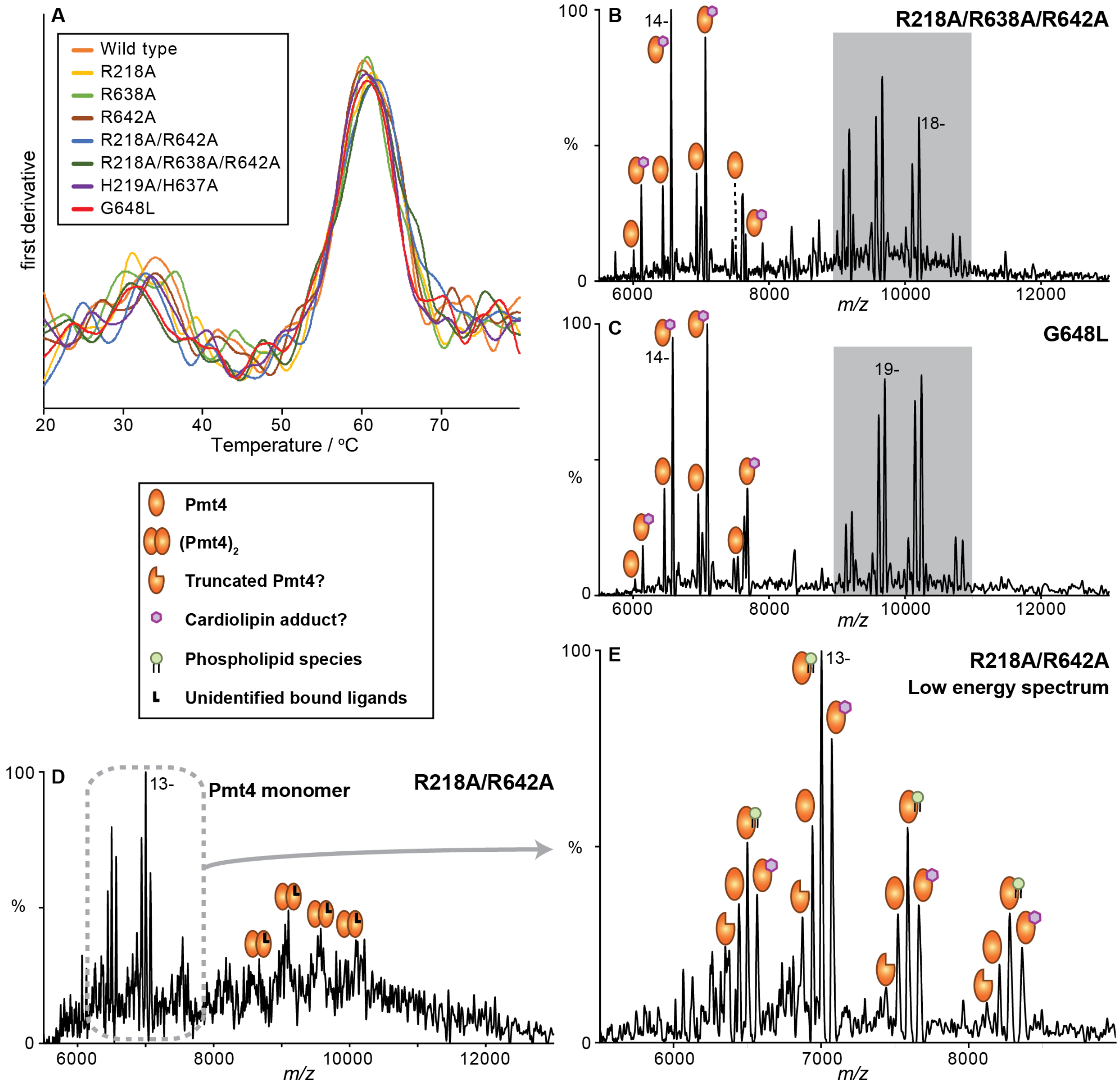

Supplementary Fig. 10. Native mass spectrometry analysis of *ct*Pmt4 mutations that disrupt Dol-P-Man binding

A) nanoDSF measurements of wild-type and Dol-P-Man binding mutants of *ct*Pmt4 at 0.3 mg/ml purified in 0.03% (w/v) DDM. Thermogram displays the first derivative of the fluorescence intensity ratio at 350 nm and 330 nm as a function of temperature. The stability of each variant as assessed by the melting temperature (T_m) at ~60°C remains unchanged. **B-E)** Mass spectra of *ct*Pmt4 mutants liberated from DDM micelles collected under non-denaturing conditions. For each assigned species, the charge state for the most intense peak is shown. Adduct peaks with a defined mass greater than the amino acid sequence mass correspond to a mixture of bound phospholipids and a putative adduct between detergent and a lipid such as cardiolipin. Adduct peaks corresponding to bound Dol-P-Man are not present.

The grey boxes in the full mass spectra of **B)** R218A/R638A/R642A and **C)** G648L mutants of *ct*Pmt4 correspond to the zoomed regions shown in Figs. 4C and 4D respectively. **D)** The resolution of the full mass spectrum for *ct*Pmt4 R218A/R642A was insufficient to assign bound ligands for either the dimeric species, probably due to protein degradation or increased complexity of lipid binding to this mutant. **E)** The adduct peaks for the *ct*Pmt4 R218A/R642A monomer could be assigned by subsequently acquiring a 'low energy' mass spectrum (i.e. by applying lower voltages in and subsequently decreasing the collision-induced dissociation of the protein-micelle complex).

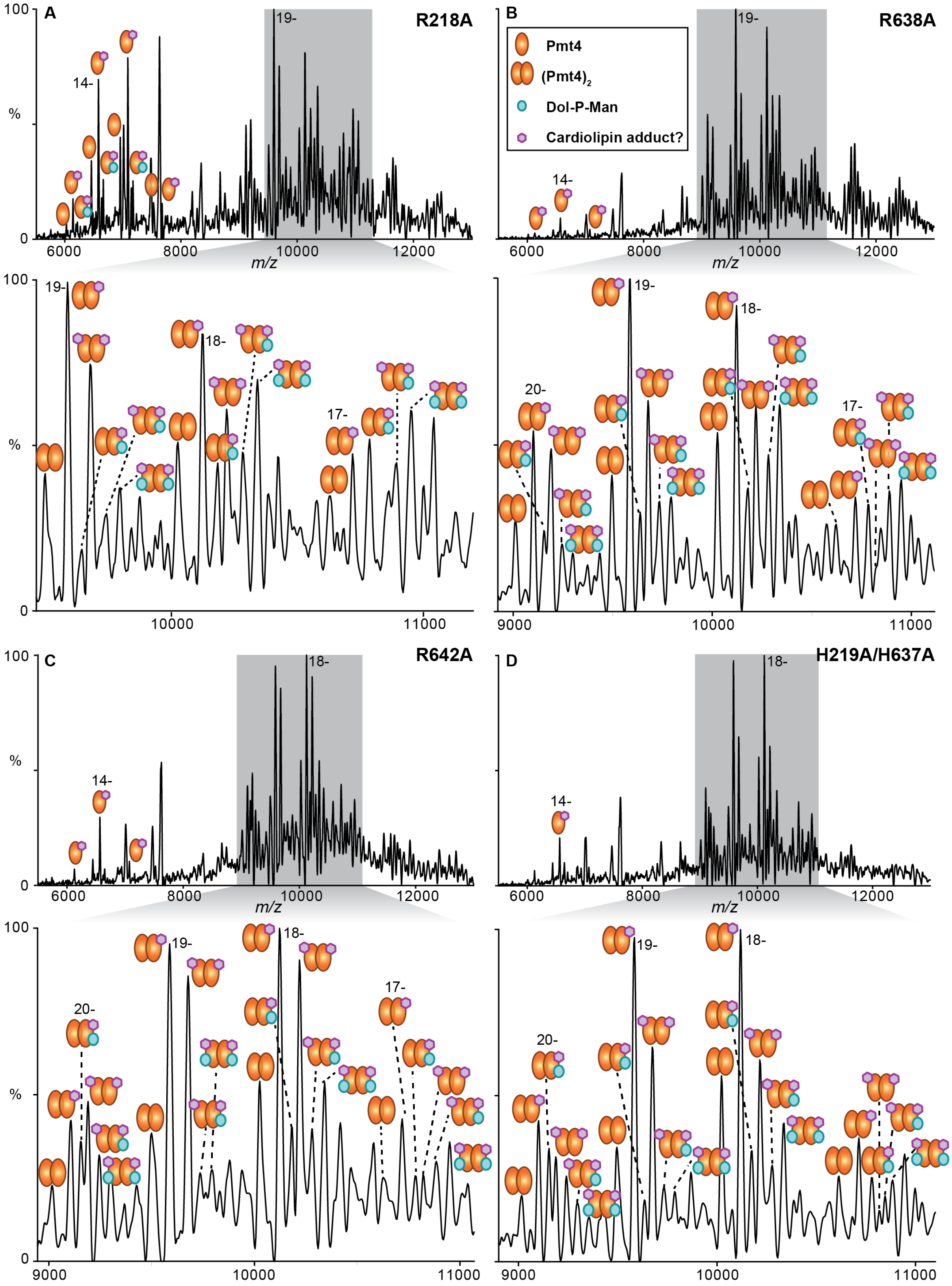

Supplementary Fig. 11. Native mass spectrometry of *ct*Pmt4 mutations that do not substantially impair Dol-P-Man binding

Mass spectra of *ct*Pmt4 mutants liberated from DDM micelles collected under non-denaturing conditions. For each assigned species, the charge state for the most intense peak is shown. The zoomed region shows the main charge states for dimeric *ct*Pmt4. Adduct peaks with a defined mass greater than the amino acid sequence mass correspond to a mixture of bound Dol-P-Man and a putative adduct between detergent and a lipid such as cardiolipin. The **A)** R218A, **B)** R638A, **C)** R642A and **D)** H219A/H637A point mutations in the cytosolic Dol-P-Man binding site do not substantially impair binding of the lipid.

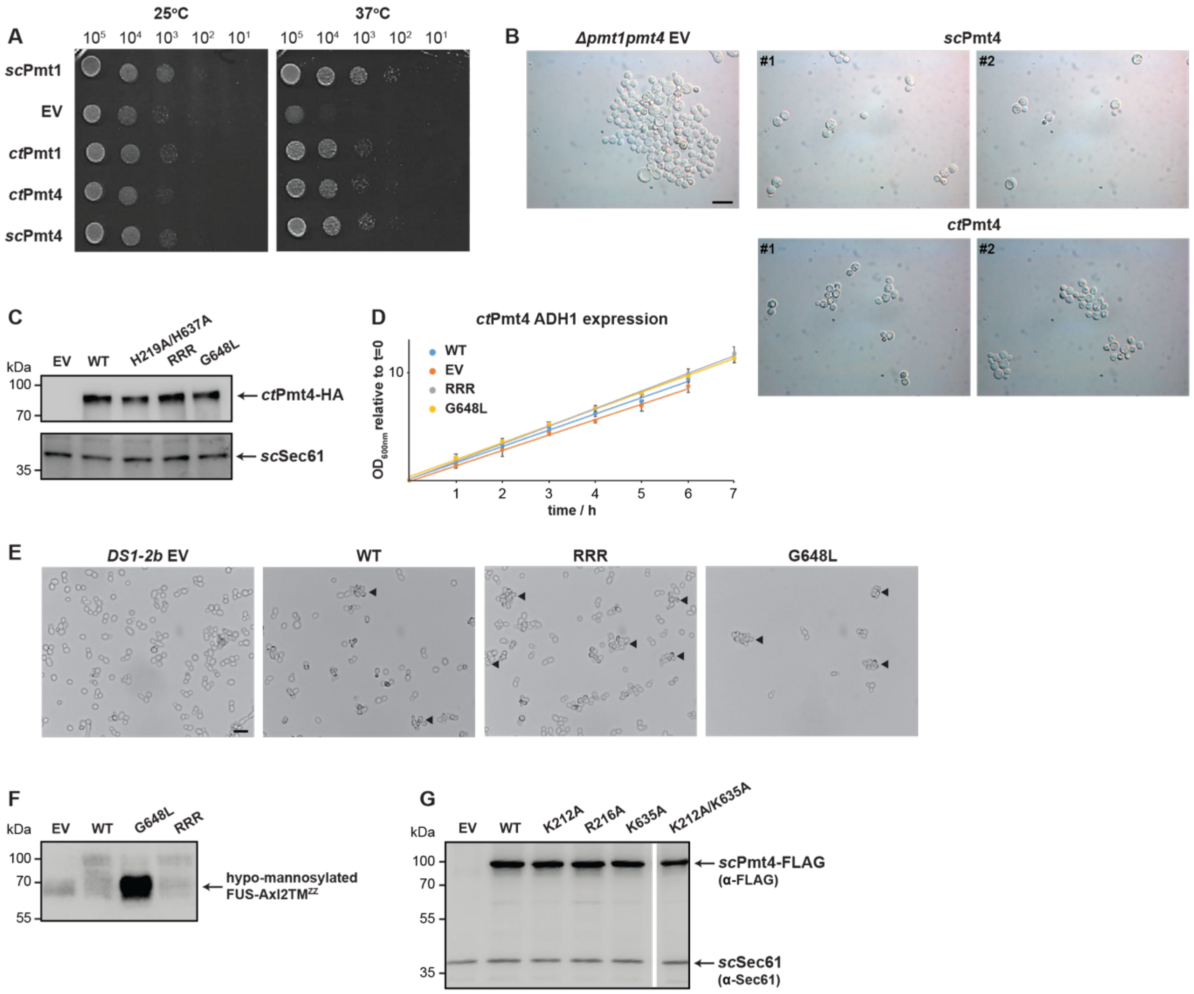

Supplementary Fig. 12. In vivo characterisation of Pmt4 mutations in the Dol-P-Man cytosolic binding pocket

A) Serial dilutions of yeast strain CFY3 (*Δpmt1pmt4*) expressing empty pREP3-adh vector (EV), *sc*Pmt1, *sc*Pmt4, *ct*Pmt1 or *ct*Pmt4 on YPD plates grown for 3 days at 25 °C and 35 °C. **B)** Light micrographs of transformed cells as in **A** grown in SC medium -Leu to the logarithmic phase (OD_{600nm} ~1). Scale bar corresponds to 30 µm. **C)** Western blot of *ct*Pmt4-HA isolated from *pmt4-G/AXL* transformed with empty pREP3-adh vector (EV), wild-type *ct*Pmt4 (WT) or Dol-P-Man binding mutants under the control of the constitutive *ADH1* promoter. 16 µg total membrane protein from each strain was resolved on a 10% SDS-PA gel and probed with anti-HA antibody. Sec61 was analysed as a loading control. A representative blot is shown from three independent experiments. **D)** Growth of the yeast *pmt4-G* strain transformed with empty pREP3-adh vector (EV), wild-type *ct*Pmt4 (WT) or Dol-P-Man binding mutants under the control of the constitutive *ADH1* promoter. Selective medium was supplemented with 2% glucose and OD_{600nm} was measured every 60 min. Average values from three independent

experiments are shown on a logarithmic scale with error bars showing the standard deviation. **E)** Light micrographs of the yeast *pmt4-G* strain transformed with empty pMT929 vector (EV), wild-type *ct*Pmt4 (WT) or Dol-P-Man binding mutants under the control of the *GAL1/GAL10* promoter. Selective medium was supplemented with 2% galactose and cells were imaged in the logarithmic growth phase. Arrows indicate clumps of cells more prevalent in the Dol-P-Man binding mutants. Scale bar corresponds to 30 µm. **F)** Western blot analysis of the reporter protein FUS-Axl2TM^{ZZ} isolated from yeast strain *pmt4-G/AXL* transformed with empty pMT929 vector (EV), wild-type *ct*Pmt4 (WT) or Dol-P-Man binding mutants overexpressed from the *GAL1/GAL10* promoter. 40 µg total membrane protein from each strain was separated on a 8% SDS-PA gel and probed with peroxidase-coupled anti-rabbit-IgG antibody. A representative blot is shown from three independent experiments. **G)** Western blot of *sc*Pmt4-FLAG isolated from *pmt4-G/CCW5* transformed with wild-type *sc*Pmt4 (pJK4-B1; WT), empty pRS423 vector (EV) and pJK4-B1 with mutations in positively charged residues in the cytosolic pocket. 20 µg total membrane protein from each strain was resolved on 8% SDS-PA gels and probed with anti-FLAG antibody. Sec61 was analysed as a loading control. A representative blot is shown from three independent experiments. Source data for **C**, **D**, **F** and **G** are provided in the Source Data file.

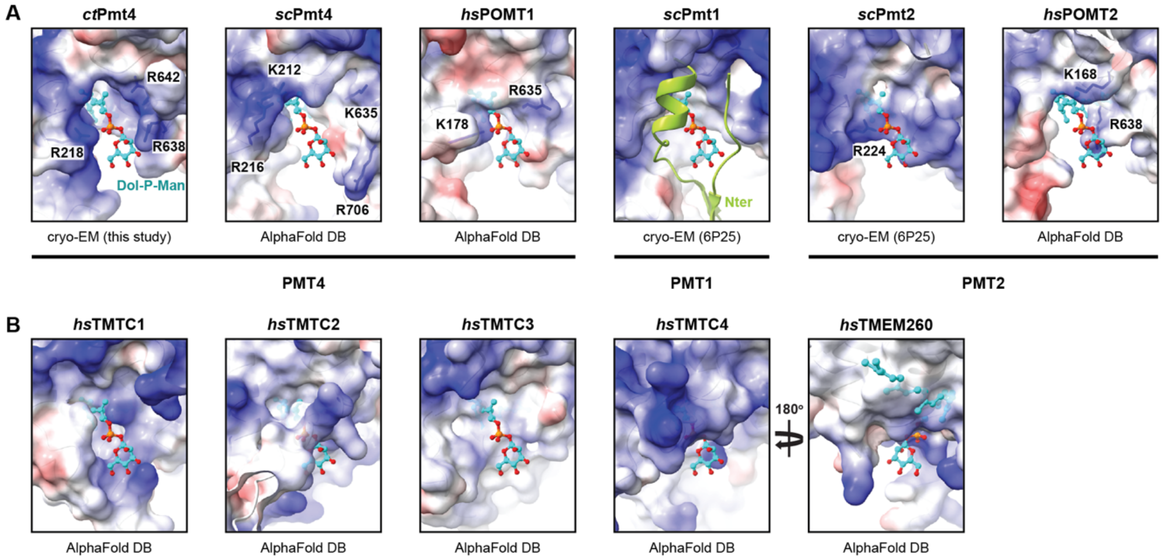

Supplementary Fig. 13. O-mannosyltransferases have a conserved positively charged cytosolic pocket

Side-by-side comparison of the cytosolic Dol-P-Man binding pocket in *ct*Pmt4 with the equivalent regions in other O-mannosyltransferases from *S. cerevisiae* and *H. sapiens*. The electrostatic surface potentials (± 10 kT) are shown and Dol-P-Man bound to *ct*Pmt4 is superimposed with each enzyme. **A)** Comparison with representative PMT homologues from the PMT4, PMT1 and PMT2 families. The TMDs of *ct*Pmt4 were superimposed with the AlphaFold DB[23,24] models of *sc*Pmt4 (RMSD 1.00 Å over 487 Cα-atoms), *hs*POMT1 (RMSD 1.09 Å over 460 Cα-atoms) and *hs*POMT2 (RMSD 1.03 Å over 453 Cα-atoms) and the cryo-EM structure (PDB 6P25) of *sc*Pmt1 and *sc*Pmt2 (as in Fig. 1C). Positively charged residues are labelled and the N-terminus of *sc*Pmt1 is shown in cartoon representation for clarity. **B)** Comparison with the human TMTC and TMEM260 O-mannosyltransferases. The TMDs of *ct*Pmt4 were superimposed with the AlphaFold DB models of *hs*TMTC1 (RMSD 1.25 Å over 78 Cα-atoms), *hs*TMTC2 (RMSD 1.45 Å over 85 Cα-atoms), *hs*TMTC3 (RMSD 1.18 Å over 44 Cα-atoms), *hs*TMTC4 (RMSD 1.23 Å over 65 Cα-atoms) and *hs*TMEM260 (RMSD 1.09 Å over 102 Cα-atoms).

Supplementary Tables

Supplementary Table 1. Molecular masses determined by native mass spectrometry

Figure	*ct*Pmt4 variant	Experimental molecular mass ± s.d. (Da)	Theoretical molecular mass (Da)
	**Wild type**		
4A	*ct*Pmt4	90,348 ± 7.6	90,256
	*ct*Pmt4 + cardiolipin adduct	92,051 ± 2.7	
	*ct*Pmt4 + cardiolipin adduct + dol-P-man	93,208 ± 5.4	
	$(ct\text{Pmt4})_2$	180,693 ± 6.0	180,512
	$(ct\text{Pmt4})_2$ + cardiolipin adduct	182,405 ± 11	
	$(ct\text{Pmt4})_2$ + cardiolipin adduct + dol-P-man	183,494 ± 19	
	$(ct\text{Pmt4})_2$ + (cardiolipin adduct)$_2$	184,130 ± 21	
	$(ct\text{Pmt4})_2$ + (cardiolipin adduct)$_2$ + dol-P-man	185,302 ± 49	
	$(ct\text{Pmt4})_2$ + (cardiolipin adduct)$_2$ + (dol-P-man)$_2$	186,390 ± 26	
	**R218A/R638A/R642A**		
S10B	*ct*Pmt4$^{R218A/R638A/R642A}$	90,131 ± 17	90,001
	*ct*Pmt4$^{R218A/R638A/R642A}$ + cardiolipin adduct	91,828 ± 27	
4C	$(ct\text{Pmt4}^{R218A/R638A/R642A})_2$	180,135 ± 77	180,002
	$(ct\text{Pmt4}^{R218A/R638A/R642A})_2$ + cardiolipin adduct	181,897 ± 29	
	$(ct\text{Pmt4}^{R218A/R638A/R642A})_2$ + (cardiolipin adduct)$_2$	183,642 ± 48	
	**G648L**		
S10C	*ct*Pmt4^{G648L}	90,454 ± 13	90,312
	*ct*Pmt4^{G648L} + cardiolipin adduct	92,148 ± 18	
4D	$(ct\text{Pmt4}^{G648L})_2$	180,909 ± 56	180,624
	$(ct\text{Pmt4}^{G648L})_2$ + cardiolipin adduct	182,649 ± 29	
	$(ct\text{Pmt4}^{G648L})_2$ + (cardiolipin adduct)$_2$	184,358 ± 44	
	**R218A/R642A**		
S10E	*ct*Pmt4$^{R218A/R642A}$ truncated?	89,343 ± 38	90,086
	*ct*Pmt4$^{R218A/R642A}$	90,242 ± 44	
	*ct*Pmt4$^{R218A/R642A}$ + phospholipid	91,035 ± 27	
	*ct*Pmt4$^{R218A/R642A}$ + cardiolipin adduct	91,946 ± 29	
S10D	$(ct\text{Pmt4}^{R218A/R642A})_2$ + unidentified bound ligands	182,029 ± 89	180,172
	**R218A**		
S11A	*ct*Pmt4^{R218A}	90,273 ± 34	90,171
	*ct*Pmt4^{R218A} + cardiolipin adduct	91,980 ± 7.0	
	*ct*Pmt4^{R218A} + cardiolipin adduct + dol-P-man	93,131 ± 16	
	$(ct\text{Pmt4}^{R218A})_2$	180,570 ± 96	180,342
	$(ct\text{Pmt4}^{R218A})_2$ + cardiolipin adduct	182,220 ± 33	
	$(ct\text{Pmt4}^{R218A})_2$ + cardiolipin adduct + dol-P-man	183,293 ± 71	
	$(ct\text{Pmt4}^{R218A})_2$ + (cardiolipin adduct)$_2$	183,900 ± 65	
	$(ct\text{Pmt4}^{R218A})_2$ + (cardiolipin adduct)$_2$ + dol-P-man	185,137 ± 32	
	$(ct\text{Pmt4}^{R218A})_2$ + (cardiolipin adduct)$_2$ + (dol-P-man)$_2$	186,184 ± 44	
	**R638A**		
S11B	*ct*Pmt4^{R638A} + cardiolipin adduct	91,933 ± 41	
	$(ct\text{Pmt4}^{R638A})_2$	180,437 ± 146	180,342
	$(ct\text{Pmt4}^{R638A})_2$ + cardiolipin adduct	182,155 ± 77	
	$(ct\text{Pmt4}^{R638A})_2$ + cardiolipin adduct + dol-P-man	183,282 ± 104	
	$(ct\text{Pmt4}^{R638A})_2$ + (cardiolipin adduct)$_2$	183,919 ± 59	
	$(ct\text{Pmt4}^{R638A})_2$ + (cardiolipin adduct)$_2$ + dol-P-man	185,033 ± 110	
	$(ct\text{Pmt4}^{R638A})_2$ + (cardiolipin adduct)$_2$ + (dol-P-man)$_2$	186,098 ± 51	

	**R642A**		
S11C	*ct*Pmt4^{R642A} + cardiolipin adduct	91,945 ± 24	
	(*ct*Pmt4^{R642A})$_2$	180,485 ± 124	180,342
	(*ct*Pmt4^{R642A})$_2$ + cardiolipin adduct	182,221 ± 33	
	(*ct*Pmt4^{R642A})$_2$ + cardiolipin adduct + dol-P-man	183,244 ± 100	
	(*ct*Pmt4^{R642A})$_2$ + (cardiolipin adduct)$_2$	183,920 ± 69	
	(*ct*Pmt4^{R642A})$_2$ + (cardiolipin adduct)$_2$ + dol-P-man	185,012 ± 62	
	(*ct*Pmt4^{R642A})$_2$ + (cardiolipin adduct)$_2$ + (dol-P-man)$_2$	186,152 ± 18	
	**H219A/H637A**		
S11D	*ct*Pmt4$^{H219A/H637A}$ + cardiolipin adduct	91,925 ± 18	
	(*ct*Pmt4$^{H219A/H637A}$)$_2$	180,413 ± 37	180,248
	(*ct*Pmt4$^{H219A/H637A}$)$_2$ + cardiolipin adduct	182,106 ± 63	
	(*ct*Pmt4$^{H219A/H637A}$)$_2$ + cardiolipin adduct + dol-P-man	183,168 ± 80	
	(*ct*Pmt4$^{H219A/H637A}$)$_2$ + (cardiolipin adduct)$_2$	183,870 ± 60	
	(*ct*Pmt4$^{H219A/H637A}$)$_2$ + (cardiolipin adduct)$_2$ + dol-P-man	184,965 ± 89	
	(*ct*Pmt4$^{H219A/H637A}$)$_2$ + (cardiolipin adduct)$_2$ + (dol-P-man)$_2$	186,020 ± 75	

Supplementary Table 2. Plasmids used in this study

Plasmid	Reference	Experiment
pMT929	22	Empty vector control for overexpression in *pmt4-G* or *pmt4-G/AXL* (Fig. 5B, Supplementary Figs. 12E and 12F).
pMT929 *ct*Pmt4	This study	Recombinant protein production for cryo-EM and native MS (Supplementary Fig. 1) and overexpression of *ct*Pmt4 in *pmt4-G* or *pmt4-G/AXL* (Fig. 5B, Supplementary Figs. 12E and 12F).
pMT929 *ct*Pmt4 (G648L, R218A, R638A, R642A, R218A/R642A, R218A/R638A/R642A, H219A/H637A)	This study	Recombinant protein production for native MS (Fig. 4, Supplementary Figs. 10 and 11) and overexpression of *ct*Pmt4 G648L and RRR variants in *pmt4-G* or *pmt4-G/AXL* (Fig. 5B, Supplementary Figs. 12E and 12F).
pET24d *ct*Pmt4 MIR & pET24d *sc*Pmt4 MIR	This study	Recombinant protein production for X-ray crystallography (Supplementary Fig. 1).
pJK4-B1 (*sc*Pmt4-FLAG)	8	Expression of *sc*Pmt4 in *Δpmt4-CCW5* (Fig. 2E and Supplementary Fig. 6A), *Δpmt1pmt4* (Supplementary Fig. 6B) or *pmt4-G/CCW5* (Fig. 5D and Supplementary Fig. 12G).
pJK4-B1 (Δ362-368, Y362A/D364A/R366A, P427A/P430A, W496A, K212A, R216A, K635A, K212A/K635A)	This study	Expression of *sc*Pmt4 variants in *Δpmt4-CCW5* (Fig. 2E and Supplementary Fig. 6A), *Δpmt1pmt4* (Supplementary Fig. 6B) or *pmt4-G/CCW5* (Fig. 5D and Supplementary Fig. 12G).
pRS423	61	Empty vector control for expression in *Δpmt4-CCW5* (Fig. 2E and Supplementary Fig. 6A), *Δpmt1pmt4* (Supplementary Fig. 6B) or *pmt4-G/CCW5* (Fig. 5D and Supplementary Fig. 12G).
pML78 (CCW5-FLAG)	25	Expression of CCW5-FLAG in *Δpmt4-CCW5* (Fig. 2E) or *pmt4-G/CCW5* (Fig. 5D).
pREP3-adh *sc*Pmt1-HA (pTW61) and *sc*Pmt4-HA (pTW62)	62	Complementation of CFY3 (Supplementary Figs. 12A and 12B).
pREP3-adh *ct*Pmt1-HA (pTJ16) and *ct*Pmt4-HA (pTJ16)	This study	Complementation of CFY3 (Supplementary Figs. 12A and 12B) and expression of *ct*Pmt4-HA in *pmt4-G/AXL* (Fig. 5A and Supplementary Fig. 12C) or *pmt4-G* (Fig. 5B and Supplementary Fig. 12D).
pREP3-adh *ct*Pmt4-HA (G648L, R218A/R638A/R642A)	This study	Expression of *ct*Pmt4-HA variants in *pmt4-G/AXL* (Fig. 5A and Supplementary Fig. 12C) or *pmt4-G* (Fig. 5B and Supplementary Fig. 12D).
pJH1 (FUS-AXL2TM^{ZZ})	17	Expression of AXL2TM^{ZZ} in *pmt4-G/AXL* (Fig. 5A and Supplementary Fig. 12F).

Supplementary Table 3. ***S. cerevisiae*** **strains used in this study**

Strain	Genotype	Reference	Experiment
DS1-2b	MATα, trp1-Δ63, his3-Δ200, ura3-52, leu2-Δ1, Gal2+	34	Recombinant protein production
Δpmt4-CCW5	MATα, his3-Δ200, leu2–3,Δ112, lys2– 801, trp1-Δ901, ura3–52, suc2-Δ9 pmt4::TRP1[2] transformed with pML78 (CCW5-FLAG)	This study	Fig. 2E and Supplementary Fig. 6A
Δpmt1pmt4	MATα, his3-Δ200, leu2–3, Δ112, lys2–801, trp1-Δ901, ura3–52, suc2-Δ9, pmt1::URA3, pmt4::TRP1	20	Supplementary Fig. 6B
CFY3 (*Δpmt1pmt4*)	MATα, ade2-1, his3-Δ200, leu-2-3, Δ112, trp1-Δ901, ura3-52, suc2-Δ9, pmt1::HIS3, pmt4::TRP1	8	Supplementary Figs. 12A and 12B
pmt4-G	Isogenic to DS1-2b except pmt4::TRP1	This study	Fig. 5B, Supplementary Figs. 12D and 12E
pmt4-G/AXL	Isogenic to *pmt4-G* transformed with pJH1 (FUS-Axl2TM^{ZZ})	This study	Fig. 5A, Supplementary Figs. 12C and 12F
pmt4-G/CCW5	Isogenic to *pmt4-G* transformed with pML78 (CCW5-FLAG)	This study	Fig.5D and Supplementary Fig. 12G

Supplementary References

58. Madeira F*, et al.* Search and sequence analysis tools services from EMBL-EBI in 2022. *Nucleic Acids Research* **50**, W276-W279 (2022).

59. Robert X, Gouet P. Deciphering key features in protein structures with the new ENDscript server. *Nucleic Acids Research* **42**, W320-324 (2014).

60. Laguerre M, Saux M, Dubost JP, Carpy A. MLPP: A Program for the Calculation of Molecular Lipophilicity Potential in Proteins. *Pharmacy and Pharmacology Communications* **3**, 217-222 (1997).

61. Christianson TW, Sikorski RS, Dante M, Shero JH, Hieter P. Multifunctional yeast high-copy-number shuttle vectors. *Gene* **110**, 119-122 (1992).

62. Willer T, Brandl M, Sipiczki M, Strahl S. Protein O-mannosylation is crucial for cell wall integrity, septation and viability in fission yeast. *Molecular Microbiology* **57**, 156-170 (2005).
