# Supplementary material for: Structural characterisation of the fungal Pmt4 homodimer
Source: Nat Commun. 2025 Dec 14;16:11134. doi: 10.1038/s41467-025-67412-1 (PMC12705809; doi:10.1038/s41467-025-67412-1)
Supplement: Supplementary file 2 — Reporting Summary [file 41467_2025_67412_MOESM2_ESM.pdf]

nature portfolio

Corresponding author(s): Irmgard Sinning and Melanie McDowell

Last updated by author(s): Nov 21, 2025

Reporting Summary

Nature Portfolio wishes to improve the reproducibility of the work that we publish. This form provides structure for consistency and transparency in reporting. For further information on Nature Portfolio policies, see our Editorial Policies and the Editorial Policy Checklist.

Statistics

For all statistical analyses, confirm that the following items are present in the figure legend, table legend, main text, or Methods section.

n/a	Confirmed	
☐	☒	The exact sample size (*n*) for each experimental group/condition, given as a discrete number and unit of measurement
☐	☒	A statement on whether measurements were taken from distinct samples or whether the same sample was measured repeatedly
☒	☐	The statistical test(s) used AND whether they are one- or two-sided<br>*Only common tests should be described solely by name; describe more complex techniques in the Methods section.*
☒	☐	A description of all covariates tested
☒	☐	A description of any assumptions or corrections, such as tests of normality and adjustment for multiple comparisons
☐	☒	A full description of the statistical parameters including central tendency (e.g. means) or other basic estimates (e.g. regression coefficient) AND variation (e.g. standard deviation) or associated estimates of uncertainty (e.g. confidence intervals)
☒	☐	For null hypothesis testing, the test statistic (e.g. *F*, *t*, *r*) with confidence intervals, effect sizes, degrees of freedom and *P* value noted<br>*Give P values as exact values whenever suitable.*
☒	☐	For Bayesian analysis, information on the choice of priors and Markov chain Monte Carlo settings
☒	☐	For hierarchical and complex designs, identification of the appropriate level for tests and full reporting of outcomes
☒	☐	Estimates of effect sizes (e.g. Cohen's *d*, Pearson's *r*), indicating how they were calculated

Our web collection on statistics for biologists contains articles on many of the points above.

Software and code

Policy information about availability of computer code

Data collection	Cryo-EM images were acquired using EPU (Thermo Fisher).
Data analysis	X-ray diffraction data were processed with XDS, AIMLESS, STARANISO, Phaser-MR and Phenix. Cryo-EM images were processed with Relion 3.1, cisTEM (version 1.0 beta), WARP, MotionCorr2 and GCTF. Local density sharpening was performed with LocScale. Molecular models were built with COOT (version 0.8.9.2) and refined using Phenix (version 1.15.2). Structures were visualised with UCSF ChimeraX (version 1.10) and PYMOL (version 2.5; Schrödinger, LLC). Sequence analysis was performed with Clustal Omega and visualised with ESPript. Native MS and lipidomics data were analysed with Xcalibur 3.0, NaViA and UniDec.

For manuscripts utilizing custom algorithms or software that are central to the research but not yet described in published literature, software must be made available to editors and reviewers. We strongly encourage code deposition in a community repository (e.g. GitHub). See the Nature Portfolio guidelines for submitting code & software for further information.

Data

Policy information about availability of data

All manuscripts must include a data availability statement. This statement should provide the following information, where applicable:
- Accession codes, unique identifiers, or web links for publicly available datasets
- A description of any restrictions on data availability
- For clinical datasets or third party data, please ensure that the statement adheres to our policy

Coordinates for the scPmt4 and ctPmt4-MIR domains have been deposited in the Protein Data Bank (PDB) under accession numbers 9FD0 and 9FD1 respectively.

The C2 and C1 cryo-EM volumes for ctPmt4 have been deposited in the Electron Microscopy Data Bank under accession codes EMD-52631 and EMD-52632 respectively, and the C2 and C1 models in the PDB under accession numbers 9I5K and 9I5L respectively. PDB 3MAL and 6P25 were used to assist structure determination and model building. The native MS and lipidomics data have been deposited to the ProteomeXchange Consortium via the PRIDE repository with the dataset identifier PXD061837. Source data are provided with this paper.

Research involving human participants, their data, or biological material

Policy information about studies with human participants or human data. See also policy information about sex, gender (identity/presentation), and sexual orientation and race, ethnicity and racism.

Reporting on sex and gender	Our study does not involve human research participants.
Reporting on race, ethnicity, or other socially relevant groupings	N/A
Population characteristics	N/A
Recruitment	N/A
Ethics oversight	N/A

Note that full information on the approval of the study protocol must also be provided in the manuscript.

Field-specific reporting

Please select the one below that is the best fit for your research. If you are not sure, read the appropriate sections before making your selection.

☒ Life sciences ☐ Behavioural & social sciences ☐ Ecological, evolutionary & environmental sciences

For a reference copy of the document with all sections, see nature.com/documents/nr-reporting-summary-flat.pdf

Life sciences study design

All studies must disclose on these points even when the disclosure is negative.

Sample size	For the cryo-EM sample, at least 12 grids were pre-screened to identify the optimal grid for data collection. 9879 movies were collected and the final C1 and C2 maps were generated from 347,202 and 210,070 particles respectively. No formal sample size calculations were performed, rather replicate numbers were chosen to be sure of reproducibility and particles contributing to the final cryo-EM map were selected as a result of the processing pipeline in Supplementary Fig. 3.
Data exclusions	For cryo-EM, poor grids were discarded, only micrographs with CTF_fit_resolution < 4Å and FOM < 0.2 were used for processing, picked particles with FOM < 0.75 were removed and only particles corresponding to homogeneous populations of the Pmt4 homodimer were selected during the classification stages of data processing.
Replication	One cryo-EM dataset was collected providing >2,000,000 particles for subsequent processing. We since collected multiple datasets from the same sample, yielding very similar maps. We show western blots and growth curves from three independent replicates. Native MS spectra were acquired multiple times for the Pmt4 homodimer. All findings were reproducible.
Randomization	For cryo-EM, particles were randomly distributed in the ice and during processing were randomly divided into two groups for refinement and resolution estimation.
Blinding	For cryo-EM, MS and functional assays, blinding is not relevant as the variables are not subjective or influenced by the experimenter.

Reporting for specific materials, systems and methods

We require information from authors about some types of materials, experimental systems and methods used in many studies. Here, indicate whether each material, system or method listed is relevant to your study. If you are not sure if a list item applies to your research, read the appropriate section before selecting a response.

Materials & experimental systems

n/a	Involved in the study
☐	☒ Antibodies
☒	☐ Eukaryotic cell lines
☒	☐ Palaeontology and archaeology
☒	☐ Animals and other organisms
☒	☐ Clinical data
☒	☐ Dual use research of concern
☒	☐ Plants

Methods

n/a	Involved in the study
☒	☐ ChIP-seq
☒	☐ Flow cytometry
☒	☐ MRI-based neuroimaging

Antibodies

Antibodies used

anti-FLAG mouse monoclonal M2 (Sigma Aldrich; F3165) 1:5000 dilution
anti-Rabbit IgG–Peroxidase goat polyclonal (Sigma Aldrich; A6154) 1:10000
anti-HA mouse monoclonal (Cell Signalling; 2362) 1:5000 dilution
anti-GAPDH rabbit polyclonal (Sigma Aldrich; A9521) 1:5000 dilution
anti-mouse rabbit polyclonal HRP conjugate (Sigma Aldrich; A9044) 1:5000
anti-Sec61a (Karin Roemisch) 1:2500 dilution

Validation

anti-FLAG mouse monoclonal M2, numerous citations on manufacturer website: https://www.sigmaaldrich.com/DE/en/search/f3165?focus=papers&page=1&perpage=30&sort=relevance&term=F3165&type=citation_search
anti-HA mouse monoclonal, numerous citations on manufacturer website: https://www.cellsignal.com/products/primary-antibodies/ha-tag-262k-mouse-mab/2362?srsltid=AfmBOorjNbB-ldyv9Ow_8oxfA_7WZTzV8TALKUqLqkDvs3Wpcg50DWad
anti-GAPDH rabbit polyclonal, numerous citations on manufacturer website: https://www.sigmaaldrich.com/DE/en/product/sigma/a9521?srsltid=AfmBOop2TnNohQokMr7nEeit6ERFYTr7Y_ZD3xczlynpg7wvzq7f7BMA

Plants

Seed stocks

Report on the source of all seed stocks or other plant material used. If applicable, state the seed stock centre and catalogue number. If plant specimens were collected from the field, describe the collection location, date and sampling procedures.

Novel plant genotypes

Describe the methods by which all novel plant genotypes were produced. This includes those generated by transgenic approaches, gene editing, chemical/radiation-based mutagenesis and hybridization. For transgenic lines, describe the transformation method, the number of independent lines analyzed and the generation upon which experiments were performed. For gene-edited lines, describe the editor used, the endogenous sequence targeted for editing, the targeting guide RNA sequence (if applicable) and how the editor was applied.

Authentication

Describe any authentication procedures for each seed stock used or novel genotype generated. Describe any experiments used to assess the effect of a mutation and, where applicable, how potential secondary effects (e.g. second site T-DNA insertions, mosiacism, off-target gene editing) were examined.
